# Effect of Linseed Oil Dietary Supplementation on Fatty Acid Composition and Gene Expression in Adipose Tissue of Growing Goats

**DOI:** 10.1155/2013/194625

**Published:** 2013-01-27

**Authors:** M. Ebrahimi, M. A. Rajion, Y. M. Goh, A. Q. Sazili, J. T. Schonewille

**Affiliations:** ^1^Department of Veterinary Preclinical Sciences, Faculty of Veterinary Medicine, Universiti Putra Malaysia, 43400 Serdang, Selangor, Malaysia; ^2^Institute of Tropical Agriculture, Universiti Putra Malaysia, 43400 Serdang, Selangor, Malaysia; ^3^Department of Animal Science, Faculty of Agriculture, Universiti Putra Malaysia, 43400 Serdang, Selangor, Malaysia; ^4^Division of Nutrition, Department of Farm Animal Health, Faculty of Veterinary Medicine, Utrecht University, P.O. Box 80151, 3508 TD Utrecht, The Netherlands

## Abstract

This study was conducted to determine the effects of feeding oil palm frond silage based diets with added linseed oil (LO) containing high **α**-linolenic acid (C18:3n-3), namely, high LO (HLO), low LO (LLO), and without LO as the control group (CON) on the fatty acid (FA) composition of subcutaneous adipose tissue and the gene expression of peroxisome proliferator-activated receptor (PPAR)**α**, PPAR-**γ**, and stearoyl-CoA desaturase (SCD) in Boer goats. The proportion of C18:3n-3 in subcutaneous adipose tissue was increased (*P* < 0.01) by increasing the LO in the diet, suggesting that the FA from HLO might have escaped ruminal biohydrogenation. Animals fed HLO diets had lower proportions of C18:1 trans-11, C18:2n-6, CLA cis-9 trans-11, and C20:4n-6 and higher proportions of C18:3n-3, C22:5n-3, and C22:6n-3 in the subcutaneous adipose tissue than animals fed the CON diets, resulting in a decreased n-6:n-3 fatty acid ratio (FAR) in the tissue. In addition, feeding the HLO diet upregulated the expression of PPAR-**γ** (*P* < 0.05) but downregulated the expression of SCD (*P* < 0.05) in the adipose tissue. The results of the present study show that LO can be safely incorporated in the diets of goats to enrich goat meat with potential health beneficial FA (i.e., n-3 FA).

## 1. Introduction

The usually high content of saturated fatty acids (SFA) in ruminant meat can increase the risk of cardiovascular diseases [1]. However, ruminant meat may also be a good dietary source of some nutrients with health benefits including some FA such as long chain (C20) polyunsaturated fatty acids (LC-PUFA) and conjugated linoleic acid (CLA) isomers [2]. The decrease of SFA and the increase of health-beneficial FA have been an important topic in ruminant meat research. The inclusion of sources of C18:3n-3 in lamb diets, such as forages [3–5], pastures [6], linseed [7, 8], or linseed oil [9, 10], had increased the concentration of n-3 PUFA in the meat. Ruminant fats are among the richest natural sources of CLA isomers, particularly of rumenic acid (CLA cis-9 trans-11), and are the main sources of these isomers in the human diet [11]. The CLA cis-9 trans-11 is produced during ruminal biohydrogenation of C18:2n-6 to stearic acid [12] and by endogenous conversion of C18:1 trans-11 by Δ9-desaturase in tissues [13]. Feeding animals with diets rich in linoleic acid (C18:2n-6) and *α*-linolenic acid (C18:3n-3) acids increased the CLA cis-9 trans-11 content of ruminant meat [3, 6, 9, 14, 15]. However, feeding linseed oil (rich in C18:3n-3) seems to be less effective in the increase of CLA cis-9 trans-11 in intramuscular fat than sunflower oil (rich in C18:2n-6) [9, 16]. Bessa et al. [9] observed that a blend of sunflower and linseed oil might be a good approach to obtain simultaneously an enrichment in n-3 PUFA and CLA in lamb intramuscular fat. 

 Both n-3 and n-6 PUFA appear to suppress the genes that encode for several enzymes, which are involved in carbohydrate and lipid metabolism, whereas saturated, trans-, and monounsaturated fatty acids (MUFA) fail to suppress [17, 18]. The PPAR are activated by FA and regulate FA uptake and oxidation in the liver [19]. The PUFA activates PPAR and stimulates peroxisomal *β*-oxidation and peroxisome proliferation [20]. The expression of the SCD gene in the ruminants is regulated by the transcription factors SREBP1 [21, 22], PPAR-*α* [22], and PPAR-*γ* [21, 22]. Furthermore, unsaturated FA are important because they play a role in the cellular activities, metabolism, and nuclear events that govern gene transcription [23]. In this sense, the dietary n-6 and n-3 PUFA, especially arachidonic acid (C20:4n-6), have been shown to repress SCD gene expression [23]. In most cases these studies have been carried out in the rodents, where lipid metabolism is different from that in ruminants. 

 This study was conducted to determine the effects of feeding oil palm frond (OPF) silage-based diets containing different levels of linseed oil on the FA composition of subcutaneous adipose tissue and the PPAR-*α*, PPAR-*γ*, and SCD gene expression in Boer goats. 

## 2. Materials and Methods

### 2.1. Animals, Diets, and Management

Twenty-one five-month-old male Boer goats weighing 13.66 ± 1.07 Kg (mean initial body weight ± standard error) were initially drenched against parasites and randomly assigned to different dietary treatment groups. Goats were housed individually in wooden pens measuring 1.2 m × 1 m each, built inside a shed with slatted flooring 0.5 meter above the ground. The experimental diets were formulated to have a high LO (HLO), low LO (LLO), and no LO as the control group (CON). The feed ingredients and composition of experimental diets are shown in Table 2. Sunflower oil (SFO) (Lam Soon Edible Oils Sdn. Bhd.) which contained 6.40% of C16:0, 3.66% of C18:0, 28.32% of C18:1n-9, 61.23% of C18:2n-6, and 0.39% of C18:3n-3 expressed as a percentage of total identified FA, palm kernel oil (PKO) (Malaysian Palm Oil Board) which contained 52.55% of C12:0, 16.75% of C14:0, 9.00% of C16:0, 2.36% of C18:0, 16.62% of C18:1n-9, and 18.47% of C18:2n-6 expressed as a percentage of total identified FA, and linseed oil (LO) (Brenntag Canada, Inc., Montreal, QC, Canada) which contained 5.15% of C16:0, 3.17% of C18:0, 16.62% of C18:1n-9, 16.12% of C18:2n-6, and 57.09% of C18:3n-3 expressed as a percentage of total identified FA were used as oil sources to incorporate different levels of C18:3n-3. The linseed oil was used as the main source of *α*-linolenic acid (C18:3n-3) while sunflower oil was used as the main source of linoleic acid (C18:2n-6). The animals were fed twice daily at 3.7% of BW (DM basis), with adjustments made weekly according to the changing body weight. Fresh OPF were chopped to 2-3 cm length and mixed with cellulase enzyme (Onozuka R-10; Yakult Ltd, Tokyo, Japan; 2 g/Kg of fresh matter) and lactic acid bacteria (*Lactobacillus plantarum* MTD1, Ecosyl, Stokesley, Yorkshire, UK) calculated to contain at least 1 × 10^6^ colonies forming units (CFU) per gram as per manufacturer's instructions and ensiled in 200-liter plastic drums for 12 weeks. The concentrations (70% DM basis) and OPF silage (30% DM basis) were mixed and offered in 2 equal meals at 0800 and 1700. The diets were adjusted to be isonitrogenous and isocaloric and to meet the energy and protein requirements of growing goats (Table 2) [24]. All the goats had free access to drinking water and a mineral block. The feeding trial lasted for 100 days with a three-week adaptation period. 

### 2.2. Chemical Analyses

Samples (500 g) of concentrate and OPF silage were collected every 7 d and stored at 4°C. Individual goat refusals of feed were weighed daily and stored at 4°C until analyzed for dry matter (DM). Concentrates and OPF silage samples were dried at 60°C for 48 h to determine the DM content, ground to pass a 1 mm screen and analyzed for crude protein (CP), ether extract (EE), ash, organic matter (OM), neutral detergent fiber (NDF), and acid detergent fiber (ADF) according to standard methods. Crude protein (CP, total nitrogen × 6.25) was determined by the method (number 990.03) of the [25]. Neutral detergent fiber (NDF) and acid detergent fiber (ADF) were determined according to van Soest et al. [26]. 

### 2.3. Measurement of FA

The total FA were extracted from oils, experimental feeds, and subcutaneous adipose tissue based on the method of [27] modified by [28] and described by [29], using chloroform : methanol 2 : 1 (v/v) containing butylated hydroxytoluene to prevent oxidation during sample preparation. The extracted fatty acids were transmethylated to their fatty acid methyl esters (FAME) using 0.66 N KOH in methanol and 14% methanolic boron trifluoride (BF_3_) (Sigma Chemical Co., St. Louis, MO, USA) according to the methods by AOAC (1990). The FAME was separated by gas liquid chromatography on an Agilent 7890A GC system (Agilent, Palo Alto, CA, USA) using a 100 m × 0.25 mm ID (0.20 *μ*m film thickness) Supelco SP-2560 capillary column (Supelco, Inc., Bellefonte, PA, USA). One microliter of FAME was injected by an autosampler into the chromatograph, equipped with a flame ionization detector (FID). The carrier gas was He, and the split ratio was 10 : 1 after injection of the FAME. The injector temperature was programmed at 250°C, and the detector temperature was 300°C. The column temperature program initiated runs at 120°C held for 5 min, increased by 2°C/min up to 170°C, held at 170°C for 15 min, increased again by 5°C/min up to 200°C, held at 200°C for 5 min, then increased again by 2°C/min to a final temperature of 235°C, and held for 10 min. The FA concentrations are expressed as percent of total identified FA. A reference standard (mix C4–C24 methyl esters; Sigma-Aldrich, Inc., St. Louis, MO, USA) and CLA standard mix (CLA cis-9 trans-11 and CLA trans-10, cis-12, Sigma-Aldrich, Inc., St. Louis, MO, USA) were used to determine recoveries and correction factors for the determination of individual FA composition.

### 2.4. Tissue Collection and RNA Extraction and Purification

Immediately after slaughtering the animals the subcutaneous adipose tissue was quickly excised and snap-frozen in liquid nitrogen and stored at −80°C until RNA extraction. 

 Total RNA was extracted from 100 mg of frozen tissue using the RNeasy lipid tissue mini kit (Cat. no. 74804, Qiagen, Hilden, Germany), and DNase digestion was completed during RNA purification using the RNase-Free DNase set (Qiagen, Hilden, Germany) according to the manufacturer's instructions. Total RNA purity was determined by the 260/280 nm ratio of absorbance readings using NanoDrop ND-1000 UV-Vis Spectrophotometer (NanoDrop Technologies, Wilmington, DE, USA). 

### 2.5. Complementary DNA Synthesis

Purified total RNA (1 *μ*g) was reverse transcribed using a QuantiTect reverse transcription kit (Qiagen, Hilden, Germany) in accordance with the manufacturer's recommended procedure. 

### 2.6. Real-Time Polymerase Chain Reaction (PCR)

Real-time PCR was performed with the Bio-Rad CFX96 Touch (Bio-Rad Laboratories, Hercules, CA, USA) using optical grade plates using QuantiFast SYBR green PCR kit (Cat. no. 204054, Qiagen, Hilden, Germany). The sequences of primers are shown in Table 1.

The *β*-actin was used as the reference gene to normalize the tested genes. All primers were purchased through 1st BASE oligonucleotide synthesis (1st Base, Singapore). Each reaction (20 *μ*L) contained 8.5 *μ*L SYBR green PCR mix, 1 *μ*L cDNA, 1 *μ*L each of forward and reverse primers, and 8.5 *μ*L RNase free water. Target genes were amplified through the following thermocycling program: 95°C for 10′, 40 PCR cycles at 95°C for 30′′, 60°C for 20′′, and 72°C for 20′′. Fluorescence was measured at every 15′′ to construct the melting curve. A real-time PCR was conducted for each primer pair in which cDNA samples were substituted with dH_2_O to verify that exogenous DNA was not present. Additionally, 1 *μ*g of RNA isolated by the procedure described above was substituted for cDNA in a real-time PCR reaction to confirm that there were no genomic DNA contaminants in the RNA samples. Both negative controls showed no amplification after 40 cycles. Efficiency of amplification was determined for each primer pair using serial dilutions. The cycle numbers at which amplified DNA samples exceeded a computer generated fluorescence threshold level were normalized and compared to determine relative gene expression. Higher cycle number values indicated lower initial concentrations of cDNA and thus lower levels of mRNA expression. Each sample was run in triplicate, and averaged triplicates were used to assign cycle threshold (CT) values. The ΔCT values were generated by subtracting experimental CT values from the CT values for *β*-actin targets amplified with each sample. The group with the highest mean ΔCT value (lowest gene expression) per amplified gene target was set to zero, and the mean ΔCT values of the other groups were set relative to this calibrator (ΔΔCT). The ΔΔCT values were calculated as powers of 2 (−2 ΔΔCT), to account for the exponential doubling of the PCR.

### 2.7. Statistical Analysis

Results were analyzed using analysis of variance with the different LO content as the main effects. FA data and all the gene expression data were analyzed by one-way ANOVA, using the MIXED procedure of the SAS software package, version 9.1 (SAS Inst. Inc., Cary, NC). The statistical models used the following equation:

(1) $$\begin{array}{c} Y_{ijk}=\mu +T_{i}+F_{k}+e_{ijk}, \end{array}$$

where *μ* was the overall mean, *T* was the different dietary LO, *F* was the animal effect, and *e* was the residual error. The random effect was the animals. Means were separated using the “PDIFF” option of the “least-squares means (LSMEANS)” statement of the MIXED procedure. Differences of *P* < 0.05 were considered to be significant. Linear and quadratic contrasts were used to determine the effect of increasing amounts of LO on the response variables. The data were checked for normality using the UNIVARIATE procedure of SAS software, and the results in the tables are presented as means ± standard error of the mean. 

## 3. Results 

### 3.1. Composition of Experimental Diets

The three experimental diets which were isocaloric are shown in Table 2. The average metabolizable energy content ranged from 2.41 to 2.51 Mcal/Kg of the dry matter (DM) content, whilst the protein (13% of DM) and crude fat content (7% of DM) of the treatment diets were also similar.

As expected the FA composition of linseed oil contained the highest amount of *α*-linolenic acid and sunflower oil contained the highest amount of linoleic acid. 

### 3.2. FA Composition of Experimental Diets

Oleic and linoleic acids were the predominant FA in all treatment diets, whereas the HLO diet showed a more balanced supply of the three main FA of plant origin, namely, oleic, linoleic, and *α*-linolenic, although the latter was quantitatively the most abundant (Table 3).

### 3.3. Adipose Tissue FA Composition

The FA composition of the goat subcutaneous adipose tissue fed different dietary LO is presented in Table 4. The proportion of subcutaneous adipose tissue FA having 18 carbons was quite consistent across the three treatment groups, averaging between 56.77 and 58.23%. Mean concentrations of C18:0, C18:1n-9, C18:2n-6, and C18:3n-3 were 7.58, 39.72, 5.52, and 0.98%, respectively. On the other hand, C18:0 increased in a linear manner with increasing LO. The different levels of dietary LO had significant (*P* < 0.05) effects on some of the polyunsaturated fatty acids (PUFA) in the subcutaneous adipose tissue of especially the HLO treatment group. However, oleic acid and other monounsaturated fatty acid (MUFA) were not affected by the different LO. The C18:1 trans-11 (vaccenic acid) (VA) showed a linear increase in the CON diet compared to the LLO and HLO diets. The CLA cis-9 trans-11 (rumenic acid) also showed a positive linear effect with decreased LO. Decreasing the LO increased the C20:4n-6 (*P* < 0.01), while the HLO diet increased (*P* < 0.01) the C18:3n-3 concentration in the subcutaneous adipose tissue when compared to the CON group.

The total n-3 PUFA content in the HLO diet significantly increased (*P* < 0.01) compared to the CON diet and the n-6 : n-3 fatty acid ratios (FAR) in the subcutaneous adipose tissue of the HLO treatment group were significantly (*P* < 0.01) decreased compared to the CON treatment group (Table 4). Incremental amounts of linseed oil in the diet resulted in dose-dependent increases in most 22-carbon PUFA in the adipose tissue. The concentrations of C22:5n-3 and C22:6n-3 were significantly (*P* < 0.01) increased by increasing the LO in the diet. 

 The total SFA, MUFA, and PUFA were not affected by different dietary LO. However total n-3 PUFA increased with increasing dietary LO. In contrast, the sum of CLA isomers showed a positive linear decrease to increasing LO in the diet. The major increase in this ratio was observed in the CON diet with the highest level of n-6 : n-3 FAR (Table 4). Increasing the dietary LO by 3.96-fold (from 13.63 to 3.44; Table 2) via oil supplementation changed by almost the same ratio by 3.31-fold in the subcutaneous adipose tissue from 1.59 to 0.48 (Table 4).

### 3.4. Adipose Tissue Gene Expression

**Figure 1:**
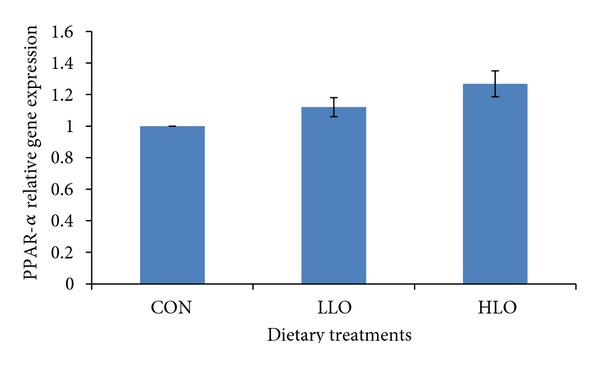
Comparison of PPAR-*α* relative gene expression in the subcutaneous adipose tissue of Boer goats fed diets with different levels of linseed oil. Values were normalized with a housekeeping gene, *β*-actin. Then, treated samples were expressed relative to gene expression of CON group. Values are means ± 1 standard error bar. HLO: high LO; LLO: low LO; CON: without LO.

**Figure 2:**
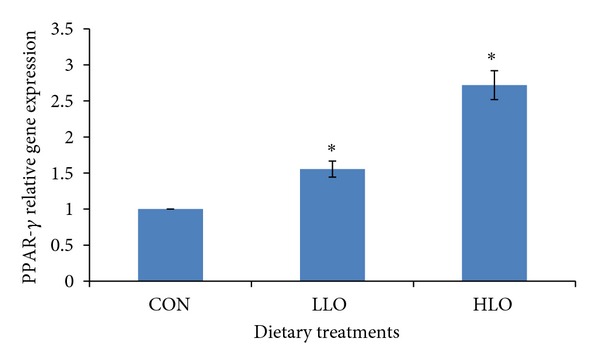
Comparison of PPAR-*γ* relative gene expression in the subcutaneous adipose tissue of Boer goats fed diets with different levels of linseed oil. Values were normalized with a housekeeping gene, *β*-actin. Then, treated samples were expressed relative to gene expression of CON group. Values are means ± 1 standard error bar. Values indicated by the ∗ show significant difference compared with the CON group (*P* < 0.05). HLO: high LO; LLO: low LO; CON: without LO.

**Figure 3:**
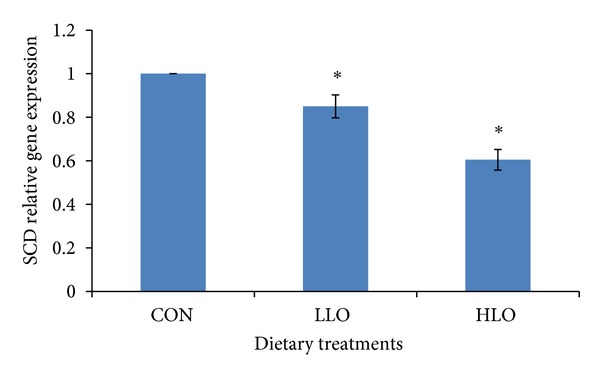
Comparison of SCD relative gene expression in the subcutaneous adipose tissue of Boer goats fed diets with different levels of linseed oil.Values were normalized with a housekeeping gene, *β*-actin. Then, treated samples were expressed relative to gene expression of CON group. Values are means ± 1 standard error bar. Values indicated by ∗ show significant difference compared with the CON group (*P* < 0.05). HLO: high LO; LLO: low LO; CON: without LO.

The relative expression of the genes in the subcutaneous adipose tissue of the HLO group compared to CON group is shown in Figures 1, 2, and 3. The PPAR-*α* gene showed a similar level of expression in all treatment groups (*P* > 0.05, Figure 1) indicating that different LO levels in the diet had no effect on the upregulation of the PPAR-*α* gene expression. The current study showed that the LO altered the PPAR-*γ* expression in the goat tissues where increasing the LO increased the PPAR-*γ* expression in the HLO and LLO treatment groups compared to the CON group. 

The SCD gene expression showed a significant (*P* < 0.05, Figure 3) reduction in the HLO group compared to the CON group suggesting that the SCD gene was downregulated by the HLO and LLO treatment. This effect was more pronounced in the LO group.

## 4. Discussion 

### 4.1. Adipose Tissue FA Composition

The FA composition of adipose tissue is determined by *de novo* lipogenesis, desaturation, dietary lipids composition and the difference in the utilization of various FA by the animal body. The proportions of the predominant FA in the goat subcutaneous adipose tissue in this study were similar to the FA proportions of the subcutaneous adipose tissue of beef heifers fed different plant oil sources as reported by Noci et al. [16].

The linear increase in subcutaneous adipose tissue concentrations of C18:1 trans-11 by decreasing dietary LO may be explained by the ability of ruminal bacteria to synthesis this isomer from C18:2n-6 [12]. Harfoot et al. [31] reported that C18:1 trans-11 was the primary end product in the rumen rather than C18:0 when C18:2n-6 was supplied in higher concentrations. The concentrations of C18:2n-6 that ranged from 4.06 to 6.76% were somewhat similar to the 4.78% reported for the subcutaneous adipose tissue of Hanwoo steers fed linseed [32] but higher than the 1.43% reported in heifers fed different vegetable oils [16]. The concentration of C18:2n-6 tended to decrease (*P* < 0.01) with increasing dietary LO, possibly because of its partial conversion to C18:0 in the rumen. The decrease in C20:4n-6 when the dietary LO was increased to 1.30% which coincided with the major decrease in dietary concentrations of C18:2n-6 was also reported by Igarashi et al. [33] for rat adipose tissue. The quadratic increases in the concentration of n-3 PUFA (C18:3n-3, C22:5n-3, and C22:6n-3) coincided with the major increase in dietary concentration of C18:3n-3 of the HLO diet. These increases are in agreement with the results of Kim et al. [34] who fed cattle with linseed. Jerónimo et al. [35] also reported that the concentration of C22:5n-3 and C22:6n-3 increased in ruminant intramuscular fat when they were fed high levels of linseed oil rather than sunflower oil. The capacity of conversion of C18:3n-3 to health promoting n-3 LC-PUFA is limited in humans [36] stressing the importance for its dietary supply. Increasing the LO in the diet resulted in a partial substitution of n-6 PUFA by n-3 PUFA in membranes. A possible reason can be the competition between C18:2n-6 and C18:3n-3 for the same desaturation and elongation enzymes which affect the conversion to LC-PUFA derivatives. Generally, a positive relationship has been reported between the concentrations of dietary C18:2n-6 and adipose tissue CLA cis-9 trans-11 in grazing heifers fed diets supplemented with plant oil-enriched concentrates [16]. The C18:1 trans-11 isomer is an intermediate product in the microbial biohydrogenation of dietary C18:1n-9, C18:2n-6, and C18:3n-3 [12]. The C18:1 trans-11 concentration in the subcutaneous adipose tissue, which could be converted to CLA cis-9 trans-11, tended to increase linearly with decreasing dietary LO. Likewise, the concentrations of CLA cis-9 trans-11 increased linearly (*P* < 0.01) as the goats consumed diets of decreasing LO, with the concentration of CLA cis-9 trans-11 increasing dramatically in goats fed the CON diet. This result may be explained by the increase in C18:2n-6 in goats fed the diet without the addition of LO, indicating that more C18:2n-6 was isomerized to CLA cis-9 trans-11 and hydrogenated to C18:1 trans-11 in the rumen of goats fed the higher C18:2n-6 leading to a higher deposition in the adipose tissue. Jerónimo et al. [35] reported that when linseed oil fed to lambs was replaced with soybean oil which contained high amounts of C18:2n-6 as the fat supplement, the concentrations of C18:1 trans-11 as well as CLA cis-9 trans-11 in intramuscular fat were increased. Therefore, the synthesize of these isomers from C18:2n-6 may have been more efficient than that from C18:3n-3. At least for forage-based diets, the well-established mainstream pathway for the ruminal biohydrogenation of C18:2n-6 is straightforward with an initial isomerization with the formation of CLA cis-9 trans-11 and its reduction to C18:1 trans-11. The C18:2n-6 supplementation would therefore result in more rumen-derived CLA cis-9 trans-11 and less diverse biohydrogenation-derived FA compared with LO. The content of CLA cis-9 trans-11 in meat decreased with increasing the dietary LO, confirming the previous results of Bessa et al. [9]. In addition Noci et al. [16] observed that the CLA cis-9 trans-11 content in intramuscular fat was higher in heifers supplemented with sunflower oil as a source of C18:2n-6 than with LO.

The n-6 : n-3 FAR is highly influenced by the FA composition of the diet fed to the animals [37]. Lowering the ratio of n-6 to n-3 FA in food products have been recommended to prevent or modulate certain diseases in humans [38]. The n-6 : n-3 FAR in food should range between 1 and 4 [39]. The n-6 : n-3 FAR found in the HLO treatment (2.07) was within this range. In the HLO treatment, only 48.51% of n-3 PUFA were n-3 LC-PUFA. This can be important considering that the health benefits of n-3 PUFA are mostly associated with the n-3 LC-PUFA as the metabolism of C18:3n-3 in humans is limited [36]. The PUFA : SFA and n-6 : n-3 FAR are indices used to evaluate the nutritional value of fat for human consumption. Increasing the PUFA content of the diet, by including sources rich in either n-6 or n-3 PUFA, generally improves the PUFA : SFA ratio [40]. This was also observed in the present trial, and in all diets where the PUFA : SFA ratio was always lower than 0.29, which is the minimum value recommended for the human diet.

### 4.2. Adipose Tissue Gene Expression

Previous studies have also revealed that the PUFA activated the PPAR efficiently, although the very LC-PUFA such as erucic acid and nervonic acid and short-chain FA (<Cl0) cannot activate the PPAR-*α* and PPAR-*γ* because these FA are exclusively metabolized in the peroxisomes [41]. The PPAR-*α* responds to changes in dietary fat by activating the expression of various enzymes involved in fatty acyl CoA formation and hydrolysis, FA elongation and desaturation, and FA oxidation [42].

The significant differences in the mRNA expression of PPAR-*γ* in the subcutaneous adipose tissue between the different dietary treatments suggest that the principal pathway through which FA act to modulate the expression of lipogenic genes is through the altered expression of PPAR-*γ*. Al-Hasani and Joost [43] also showed that increasing the dietary LO in the rodent diet can increase PPAR-*γ* activity in target tissues which is associated with increased insulin sensitivity. An alternative explanation is that the effects of FA on PPAR-*γ* signaling are mediated through changes in PPAR-*γ* activity rather than changes in gene expression, which was not measured in this study, since FA have been shown to act as natural ligands of the PPAR-*γ* gene in other studies [44]. This study also showed that the PPAR-*γ* contributes to the regulation of subcutaneous adipose tissue gene expression. As PPAR-*γ* is related to the expression regulation of several gene-encoding proteins involved in adipocyte metabolism, it could also be a candidate gene affecting the fat deposition, including intramuscular and subcutaneous adipose tissue deposition [45]. The results of the current study clearly support that there is a relationship between gene expression of PPAR-*γ* and the intake of the n-3 PUFA. The increase in n-3 PUFA of the adipose tissues in the present study increased the PPAR-*γ* gene expression. Increasing the dietary LO might stimulate PPAR-*γ* target gene expression such as lipoprotein lipase (LPL), fatty acid transport protein (FTTP), and acyl-CoA synthase (ACS) [46, 47].

To the best of our knowledge, this is the first study that examined the effects of different dietary LO levels on the PPAR-*α*, PPAR-*γ*, and SCD mRNA expression in goat tissues. This study demonstrated for the first time that the dietary LO inhibits the expression of the gene that codes for the critical enzyme required to desaturate vaccenic acid to CLA in the goat adipose tissue. Furthermore, there is evidence from the present study that the degree of inhibition of transcription for this gene was related to the dietary LO level. 

The expression of SCD is known to be strongly modulated by several nutrients such as FA, carbohydrates and hormones [18, 22, 48], and cholesterol [49]. Similarly, Daniel et al. [50] also showed a reduction in SCD gene expression in the adipose and liver tissues of lambs fed forage compared with a concentrate-based diet. The downregulation in mRNA levels was probably due to the high concentration of C18:3n-3 in the forage compared with the concentrate diet. Alpha-linolenic acid (C18:3n-3) also inhibited SCD gene expression in the mouse adipocytes [18, 51]. Given that C18:3n-3 is the predominant essential FA in grass [52], these results have implications for strategies to further augment concentrations of CLA in the tissue of goats reared on pasture. Waters et al. [22] and Igarashi et al. [18] found a negative relationship between SCD gene expression and n-3 PUFA in beef cattle and the rat, respectively, which is supported by the present finding where downregulation of SCD occurred for the HLO dietary treatment group with the highest concentration of *α*-linolenic acid. Bellinger et al. [53] showed that feeding a mixture of n-3 PUFA, *α*-linolenic acid (eicosapentaenoic acid), EPA, and docosahexaenoic acid (DHA) resulted in a 50% suppression of SCD mRNA in the rat liver. Nutrients, especially FA, have been shown to regulate SCD at both the enzyme activity [51] and transcriptional level [54]. In human cell lines, the transcription of SCD is under the control of two transcription factors, namely, PPAR-*α* and PPAR-*γ* [54]. The present study also examined the gene expression of these two transcription factors as affected by the different dietary LO, and while there was downregulation of the SCD gene expression by increasing the dietary LO, the PPAR-*γ* gene expression was significantly increased in the adipose tissue. Actually, the PPAR is a key regulator of SCD which mediates its transcriptional activation [55].

Nutritionists strongly recommend an increased human consumption of CLA and n-3 PUFA [56–58]. The CLA in human tissues may be synthesized through the tissue desaturation of vaccenic acid by SCD [59] and thus may be increased by vaccenic acid in the human diet. Results of the present study have important implications with regards to ingesting n-3 PUFA which may have negative effects on the de novo synthesis of CLA in the human muscle through potential reductions in the SCD gene expression as shown in this and many other studies. However, because a positive relationship of n-6 PUFA and particularly the n-6 : n-3 FAR with SCD gene expression exists, a correct balance of dietary n-3 PUFA appears to be of critical importance to achieve optimal SCD gene expression levels and, in turn, CLA production in the adipose tissue. A ruminant product that naturally contains CLA and n-3 PUFA could be a good regular source of these important FA and a good alternative to more expensive nutritional supplements. The present study has important implications for the establishment of dietary strategies to augment the concentration of both CLA and n-3 PUFA in ruminant tissues. However, further work is required to determine the biochemical and molecular mechanisms controlling the synthesis and deposition of n-3 PUFA and CLA in the goat tissues to optimize the effects of n-3 PUFA on the PPAR-*α*, PPAR-*γ*, and SCD gene expression. This will provide better strategies to consistently produce nutritionally enhanced chevon.

## 5. Conclusion 

 Increasing the linseed oil in the goat diet had increased the total n-3 PUFA due to the high levels of C18:3n-3 in linseed oil, resulting in a reduced n-6 : n-3 FAR of the subcutaneous adipose tissue. However, the synthesis of EPA, docosapentaenoic acid (DPA), and DHA from dietary C18:3n-3 seems to be limited, and thus the EPA, DPA, and DHA enriched goat meat would contribute only a small amount compared to the recommended daily intake for human diet. The results indicate that maximum of tissue CLA cis-9 trans-11 concentration was observed with the low C18:3n-3 in the diet which contained low amounts of linseed oil and it decreased linearly by increasing the C18:3n-3.

 The present study investigated how changes in the dietary FA affect the mRNA level expression of genes related to fat metabolism in the subcutaneous adipose tissue in Boer goats. The results showed that different dietary fat led to different FA profiles in the adipose tissues and levels of PPAR-*α*, PPAR-*γ*, and SCD gene expression. Goats fed treated OPF-based diets with high n-3 PUFA showed an upregulation of the PPAR-*γ* and downregulation of the SCD gene expression compared to the goats fed with low n-3 PUFA.

Thus, the data indicate that utilization of diets with the highest *α*-linolenic acid is a valid approach to obtain goat meat enriched with n-3 PUFA. Increasing the n-3 PUFA in the diet using linseed oil decreased the n-6 : n-3 FAR in the subcutaneous adipose tissue of growing Boer goats. These changes would likely improve the health status of the chevon produced.

## Figures and Tables

**Table 1:** Names and sequences of the primers used in this study.

Target group		Sequence 5′-3′	Length, nt	Reference
*β*-actin	F	CGC CAT GGA TGA TGA TAT TGC3	123	[22]
	R	AAG CGG CCT TGC ACA T3		
PPAR-*α*	F	TGC CAA GAT CTG AAA AAG CA	101	[30]
	R	CCT CTT GGC CAG AGA CTT GA		
PPAR-*γ*	F	CTT GCT GTG GGG ATG TCT C	121	[30]
	R	GGT CAG CAG ACT CTG GGT TC		
SCD	F	CCC AGC TGT CAG AGA AAA GG		
	R	GAT GAA GCA CAA CAG CAG GA	115	[30]

^1^F: forward; ^2^R: reverse.

**Table 2:** Ingredients and chemical composition of the experimental diets.

	Treatment diets		
	HLO	LLO	CON
Ingredients (% DM)			
OPF silage	30.00	30.00	30.00
Corn, grain	17.00	17.00	17.00
Soybean meal	13.30	13.30	13.30
Palm kernel cake	25.11	25.11	25.11
Rice bran	8.18	8.18	8.18
Linseed oil	1.30	0.40	0.00
Palm kernel oil	0.10	1.00	1.10
Sunflower oil	2.00	2.00	2.30
Mineral premix	0.50	0.50	0.50
Vitamin premix	0.50	0.50	0.50
Ammonium chloride	1.00	1.00	1.00
Limestone	1.00	1.00	1.00
Chemical composition			
ME (Mcal/Kg DM)^1^	2.51	2.51	2.51
CP (% DM)	13.00	13.00	13.00
EE (% DM)	7.00	7.00	7.00
NDF (% DM)	48.90	48.90	48.90
ADF (% DM)	33.00	33.00	33.00
CA (% DM)	0.68	0.68	0.68
P (% DM)	0.36	0.36	0.36

HLO: high LO; LLO: low LO; CON: without LO.

^1^Calculated values.

**Table 3:** Fatty acid composition (percentage of total identified fatty acids) of the experimental diets^1^.

Fatty acids	Treatment diets		
	HLO	LLO	CON
C10:0, capric	0.53	0.90	0.90
C12:0, lauric	3.53	7.02	7.24
C14:0, myristic	1.79	3.83	3.00
C16:0, palmitic	15.65	15.36	16.00
C16:1n-7, palmitoleic	0.21	0.24	0.22
C17:0, margaric	0.29	0.28	0.29
C18:0, stearic	5.68	5.88	5.85
C18:1n-9, oleic	27.78	27.62	27.40
C18:2n-6, linoleic	30.92	32.40	35.68
C18:3n-3, linolenic	13.63	6.47	3.44
SFA^2^	27.18	32.99	32.97
UFA^3^	72.53	66.73	66.74
MUFA^4^	20.45	27.99	27.86
n-3 PUFA^5^	13.63	6.47	3.44
n-6 PUFA^6^	30.92	32.40	35.68
n-6 : n-3 FAR^7^	2.27	5.01	10.38

HLO: high LO; LLO: low LO; CON: without LO.

^1^The data are expressed as the percentage of total identified fatty acids.

^2^SFA = sum of C10:0 + C12:0 + C14:0 + C16:0 + C17:0 + C18:0.

^3^UFA = sum of C16:1 + C18:1n-9 + C18:2n-6 + C18:3n-3.

^4^MUFA = sum of C16:1 + C18:1n-9.

^5^PUFAn-3 = sum of C18:3n-3.

^6^PUFAn-6 = sum of 18:2n-6.

^7^n-6 : n-3 FAR = (C18:2n-6) ÷ (C18:3n-3).

**Table 4:** Fatty acid composition (percentage of total identified fatty acids) of the subcutaneous adipose tissue in Boer goats fed diets with different levels of linseed oil^1^.

	Treatment diets				*P* value	
Fatty acids				SEM		
	HLO	LLO	CON		Linear	Quadratic
C10:0, capric	0.13	0.13	0.12	0.01	0.744	0.890
C12:0, lauric	2.96	3.52	3.66	0.26	0.661	0.051
C14:0, myristic	5.80	4.59	5.15	0.19	0.506	0.793
C14:1, myristoleic	0.73	0.57	0.57	0.02	0.066	0.086
C15:0, pentadecanoic	0.73	0.81	0.90	0.07	0.368	0.738
C15:1, pentadecenoic	0.17	0.27	0.29	0.03	0.710	0.110
C16:0, palmitic	23.39	22.55	20.54	0.32	0.493	0.008
C16:1n-7 palmitoleic	3.43	3.79	4.40	0.20	0.078	0.418
C17:0, margaric	1.11	1.24	1.19	0.09	0.810	0.609
C17:1, margaroleic	0.95	0.89	0.83	0.06	0.200	0.213
C18:0, stearic	7.97	7.48	7.28	0.31	0.349	0.646
C18:1n-9, oleic	39.76	40.17	39.23	0.38	0.274	0.145
C18:1trans-11, vaccenic	2.67	2.79	3.25	0.10	0.014	0.171
C18:2n-6, linoleic	4.06	5.72	6.76	0.27	0.004	0.001
CLA cis-9, trans-11	0.62	0.77	1.05	0.06	0.007	0.002
CLA cis-12, trans-10	0.10	0.13	0.17	0.01	0.031	0.013
C18:3n-3, *α*-linolenic	1.59	0.86	0.48	0.10	0.001	0.005
C20:4n-6, arachidonic	2.34	2.88	3.55	0.12	0.002	0.001
C20:5n-3, eicosapentaenoic	0.27	0.11	0.08	0.03	0.523	0.882
C22:5n-3, docosapentaenoic	0.55	0.37	0.26	0.03	0.132	0.002
C22:6n-3, docosahexaenoic	0.68	0.37	0.20	0.05	0.004	0.001
SFA^2^	42.09	40.32	38.85	0.48	0.983	0.114
UFA^3^	57.91	59.68	61.15	0.48	0.913	0.214
MUFA^4^	47.69	48.47	48.58	0.48	0.234	0.249
n-3 PUFA^5^	3.10	1.72	1.03	0.19	0.004	0.001
n-6 PUFA^6^	6.40	8.60	10.31	0.32	0.002	0.001
Total CLA^7^	0.72	0.89	1.23	0.07	0.008	0.002
n-6 : n-3 FAR^8^	2.07	5.02	10.03	0.79	0.001	0.001
UFA : SFA	1.38	1.48	1.57	0.03	0.981	0.252
PUFA : SFA ratio	0.23	0.26	0.29	0.01	0.062	0.494

HLO: high LO; LLO: low LO; CON: without LO.

^1^The data are expressed as the percentage of total identified fatty acids.

^2^SFA = sum of C10:0 + C12:0 + C14:0 + C15:0 + C16:0 + C17:0 + C18:0.

^3^UFA = sum of C14:1 + C16:1 + C17:1 + C18:1n-9 + C18:2 + C18:3 + C20:4, C22:6, C20:5n-3 + C22:5-3 + C22:6n-3.

^4^MUFA = sum of C14:1 + C16:1 + C17:1 + C18:1n-9.

^5^n-3 PUFA = sum of C18:3n-3 + C20:5n-3 + C22:5n-3 + C22:6n-3.

^6^n-6 PUFA = sum of 18:2n-6 + 20:4n-6.

^7^Total CLA = sum of CLA cis-9 trans-11 + CLA cis-12 trans-10.

^8^n-6 : n-3 FAR = (C18:2n-6 + C20:4n-6) ÷ (C18:3n-3 + C20:5n-3 + C22:5n-3 + C22:6n-3).
